# Design of a miniaturized dual circularly polarized implantable antenna by using characteristic mode method

**DOI:** 10.1038/s41598-024-67027-4

**Published:** 2024-07-16

**Authors:** Zhiwei Song, Xiaoming Xu, Yuchao Wang, Youwei Shi, Xianren Zheng, Lu Wang

**Affiliations:** 1 School of Electrical Engineering, Xinjiang University, Wulumqi, 830046 China; 2 Microsystem Center, The 58th Research Institute of China Electronics Technology Group Corporation, Wuxi, 214125 China

**Keywords:** Biotelemetry, Circular polarization, Characteristic mode analysis method, Implantable antenna, Engineering, Electrical and electronic engineering

## Abstract

The characteristic mode method is used to design a miniaturized dual-band dual circularly polarized (CP) implantable antenna operating in ISM bands. The miniaturization and dual-band characteristics are gained by using the slotting method and by inserting a short-circuit probe between the radiation patch and the ground plane. We use the characteristic mode method to study the current distribution of circular radiation patches with T-shaped slots in different modes. After opening a cross-shaped slot at the center of the radiation patch and the ground plane, we obtained two orthogonal modes with equal amplitude and phase difference of 90° in two operating frequency bands, ultimately achieving CP characteristics of the antenna. Its overall size is only $\pi$×(0.014 $\uplambda$_0_)^2^ × 0.0027 $\uplambda$_0_, smaller than other CP implantable antennas with similar performances, and it has satisfactory radiation efficiency and gain characteristics. Measurements show that it can operate in the ISM bands of 0.9 and 2.4 GHz with an effective 3 dB axial ratio bandwidth greater than 220 MHz (0.87 to 1.09 GHz, 22.45%) and 230 MHz (2.31 to 2.54 GHz, 9.48%), and its peak gain is − 29.5 dBi and − 19.2 dBi, respectively. And, this design complies with IEEE safety guidelines.

Since Nowadays, with the rapid development of wireless communication and electronic technology, the use of wireless biomedical equipment can improve the level of the medical service industry^1,2^. Such as brain implant technology, retinal prosthesis, and capsule endoscopy are the main trends in the medical service industry’s current and future development^3–5^. Some technical challenges are involved in the design of implantable antennas such as miniaturization, specific absorption rate (SAR), polarization mismatch, and generation of human body models, etc^6,7^. Two commonly used miniaturization methods are extending the current path and using a high dielectric constant substrate^8–13^. In Ref.^10^, Rogers RO 3010 (ε_r_ = 10.2) is used as the substrate while a simple slotting method is carried out to extend the current path to reduce the size of the antenna. In addition, implantable devices work inside the human body and some scholars have conducted antenna studies to extend the lifetime of such devices^14–24^. Some scholars have designed dual-band antennas in which the low-power consume in the high frequency band is used to wake up the device while the high-power consume in the low frequency band is used for transmitting data, which extends the lifetime of implantable medical devices^17,22^.

Due to the frequent movement or posture changes of the human body, linearly polarized implantable antennas are prone to polarization mismatch problems. Therefore, several scholars have been devoted to the study of circularly polarized (CP) implantable antennas. A compact CP implantable antenna for wireless health monitoring microsystems has been proposed in the literature^25^, in which the CP characteristics are obtained by cutting four L-shaped slots in the radiating patch. In addition, a method for designing circularly polarized antennas using eigenmode analysis is also proposed^26–29^. The advantage of this method is that it can deeply understand the electromagnetic behavior and carry out optimized design, but its disadvantage is that it is highly complex and requires reliance on computing resources and special tools. In Ref.^27^, a four-port multiple-input multiple-output (MIMO) circularly polarized antenna with good isolation characteristics is studied. The first six different modes of an asymmetric square groove with inverted L-shaped bars were analyzed using the eigenmode analysis (CMA). In conclusion, miniaturized multi-band CP implantable antennas can better meet the application requirements of wireless healthcare services, but the design difficulty is relatively high, especially when designing multiple bands with CP characteristics.

In this paper, a miniaturized dual-band dual-CP implantable antenna for biometric communication is designed by using characteristic mode method. The application of characteristic mode analysis can help to optimize the design of the antenna, improve the radiation characteristics, enhance the performance and simplify the design difficulty. We investigate the current distribution of a circular radiating patch with T-shaped slots in different modes using a characteristic pattern analysis method. After cutting a cross-shaped slot in the central of the radiating patch and the ground plane, respectively, we obtained two orthogonal modes with equal amplitudes in the two operating frequency bands. Its size is only 61.7 mm^3^, which is smaller than other antennas with similar radiation characteristics. Measurements show that the effective 3 dB axial ratio (AR) bandwidths are greater than 220 MHz (0.87 to 1.09 GHz, 22.45%) and 230 MHz (2.31 to 2.54 GHz, 9.48%), with peak gains of -29.5 dBi and -19.2 dBi at frequencies of 915 MHz and 2.45 GHz.

## Topology and design strategy

### Geometry of the antenna

**Figure 1:**
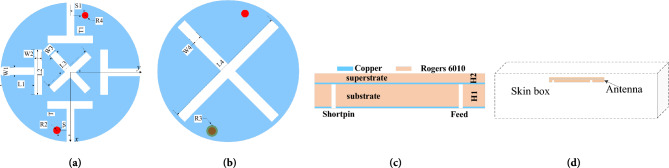
The antenna and its simulation environment. (**a**) Radiation patch with four open T-slots and a cross slot. (**b**) Ground plane with a cross slot. (**c**) Side view. (**d**) Simulation environment.

The geometry of the antenna is shown in Fig. 1. The antenna consists of a circular superstrate, a circular radiation patch with four T-shaped slots and a cross-shaped slot, a circular dielectric substrate, a circular ground plane with a cross-shaped slot, a short-circuit probe and a co-axial feed. The superstrate prevents the radiation patch from direct contact with human tissue. The radiation patch and the ground plane are located on the dielectric substrate’s upper and lower surfaces. We use Rogers RT 6010 (ε_r_ = 10.2, tanδ = 0.0023) and slotting techniques to achieve miniaturization. By cutting four T-shaped slots on the radiation patch, the effective length of the current path is increased, and the antenna size is reduced.

The exact dimensions of the antenna parameters are listed in Table 1. The dual-band characteristic is obtained by inserting a shorting probe at the axisymmetric position of the feed port between the radiation patch and GND. To further increase the bandwidth, the thickness of the dielectric substrate can be adjusted appropriately. Next, two cross slots of different sizes are cut on the radiator and the ground, which can increase the degree of freedom to adjust the antenna operating frequency and 3 dB AR bandwidth, and cover 915 MHz and 2.45 GHz of the ISM band very well. The total size is $\pi$×(0.014 $\lambda$_0_) ^2^ × 0.0027 $\lambda$_0_.

**Table 1:** Geometric parameters optimized for the proposed antenna (unit: mm).

Parameters	Value	Parameters	Value	Parameters	Value
W1	0.4	L1	2.3	S	0.7
W2	0.4	L2	3.0	T	4.0
W3	0.4	L3	4	S	0.8
W4	0.7	L4	8.8	T	4.0
R	4.7	R2	0.2	R3	0.3
R4	0.15	H1	0.635	H2	0.254

### Simulation setup and environment

The simulation analysis is carried out on the antenna implanted into a single layer of human skin tissue, as shown in Fig. 1d. The size of the simulated environment is 40 × 40 × 10 mm^3^, and the electrical characteristics of the simulated environment are the same as those of human skin tissue. In the 0.9 GHz frequency band, the conductivity is 0.87 S/m, the dielectric constant is 41.3, and in the 2.45 GHz frequency band, the conductivity is 1.464 S/m, and the dielectric constant is 38.0^30^. The antenna is set at the center of the single-layer skin tissue model. The implantation depth is 4 mm.

**Figure 2:**
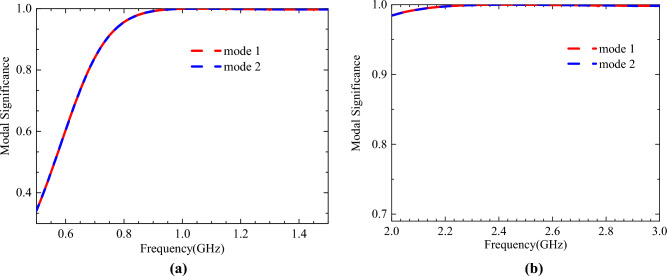
Modal significance as a function of frequency. (**a**) 0.9 GHz frequency band. (**b**) 2.4 GHz frequency band.

**Figure 3:**
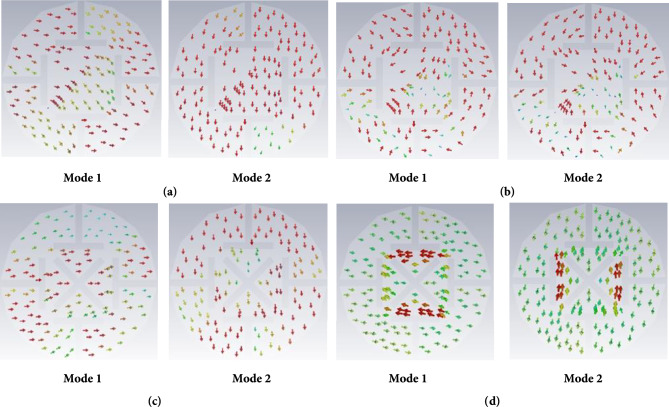
Modal significance as a function of frequency. (**a**) 0.9 GHz frequency band. (**b**) 2.4 GHz frequency band. (**c**) 0.9 GHz (with cross slot), (**d**) 2.45 GHz (with cross slot).

The characteristic mode method is carried out by using CST. The characteristic modal analysis can be used to analyze and optimize the vibration modes and radiation characteristics of the antenna. Based on the results of the mode analysis and CP analysis, the antenna design is optimized. Figure 2 shows the modal significance (MS) curve obtained from the simulation as a function of frequency. The cross slots on the radiation patch have very little effect on the modal significance and only the modal significance curves after cutting the cross slots are shown here. There are two modes with MS values equal to 1 at 0.9 GHz and 2.4 GHz, respectively. The surface current vector distribution on the radiating patch is shown in Fig. 3.

From Fig. 3a,b, it can be seen that the phase difference of the antenna surface currents is not equal to 90° in these two modes at 0.9 and 2.4 GHz, respectively. Therefore, no CP characteristics are obtained. At 0.9 GHz, the current vector direction of mode 1 is -45° (Fig. 3a). At 2.45 GHz, the current vector direction of mode 1 is about 45° (Fig. 3b). Therefore, we inserted a 45° rectangular slot and a -45° rectangular slot to cut the current path (a cross-shaped slot). At this time, the surface current vector distribution of the radiation patch is shown in Fig. 3c,d, respectively. The current vectors in the two modes are equal in amplitude and orthogonal to each other, and the CP characteristics can be achieved by simply designing a suitable feed structure.

**Figure 4:**
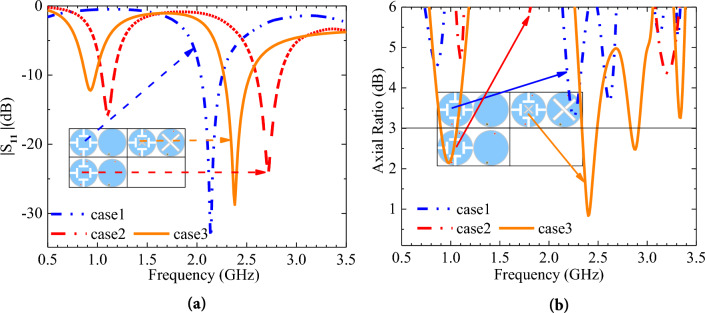
Simulated S_11_ and AR of different cases. (**a**) S_11_. (**b**) AR.

In conjunction with the characteristic mode analysis, we show three evolutionary scenarios, as shown in Table 2. In case 1, we cut four T-slots in the radiation patch. In case 2, we add a short probe. In case 3, we cut cross slots in the radiation patch and GND. The simulated S11 and AR for the three different cases are shown in Fig. 4. The conclusions are: (a) Comparison of S11 of case 1 and case 2 shows that adding a short probe causes the central frequency point moves to a lower frequency band and generates a new operating frequency band. (b) Comparison of S11 of case 2 and case 3 shows that adding a cross slot on the radiation patch causes the central frequency point moves to a lower frequency band. A comparison of AR for case 2 and case 3 shows that the addition of cross slots on the radiation patch and GND result in an AR that meets the design goals.

**Table 2:** Evolution process of antenna radiation patch and ground plane.

Case	1	2	3
Top view			
Bottom view

### Simulation analysis of critical parameters

**Figure 5:**
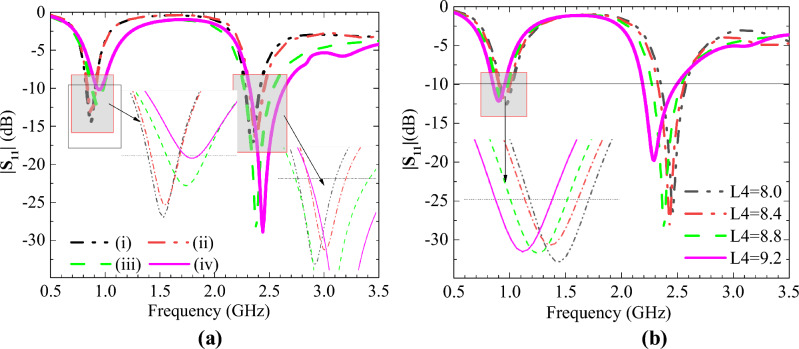
The simulated S_11_ when one parameter changes. (**a**) i:H1 = H2 = 0.254; ii:H1 = 0.254, H2 = 0.635; iii: H1 = 0.635, H2 = 0.254; iv:H1 = H2 = 0.635. (**b**) L4 with different values.

**Figure 6:**
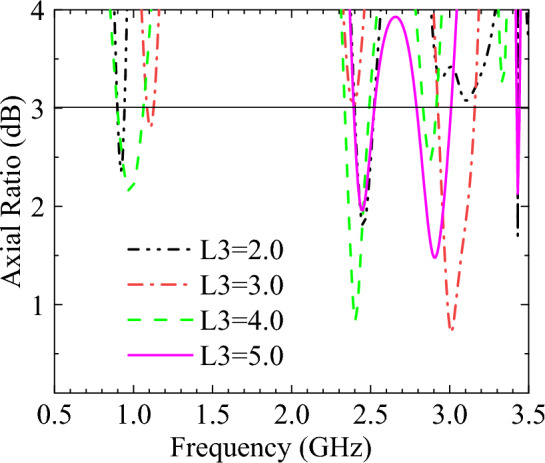
The simulated AR when L3 with different sizes.

The parameters of the designed antenna are studied, and this section describes the effect of some key parameters on the antenna impedance matching and CP characteristics. By changing some parameters, the antenna can be easily tuned and matched to the desired frequency band, and we get the following conclusions. The H1 and H2 changes affect the magnetic field distribution, and the appropriate changes in H1 and H2 can improve the impedance matching of the antenna, as shown in Fig. 5a. A constant change in the equivalent capacitance is produced due to the uniform increase in the slot length on the GND. Therefore, a sizeable homologous shift is observed in the two frequencies, as shown in Fig. 5b. The cross slots on the radiation patch and GND change the surface current distribution. This design couples energy between the radiation patch and GND, causing resonance and affecting the AR of the antenna, as shown in Fig. 6.

### Simulation results analysis

**Figure 7:**
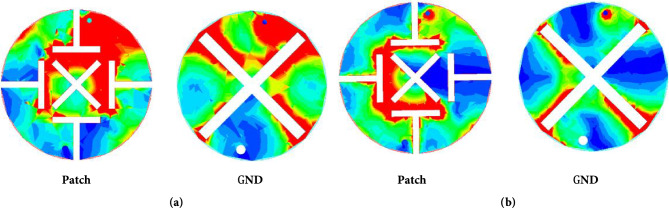
The simulated current distribution. (**a**) 0.9 GHz. (**b**) 2.45 GHz.

**Figure 8:**
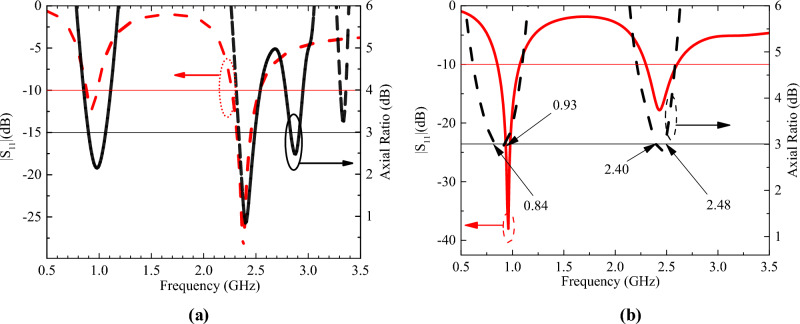
The simulated S_11_ and AR. (**a**) 0.9 GHz. (**b**) 2.45 GHz.

**Figure 9:**
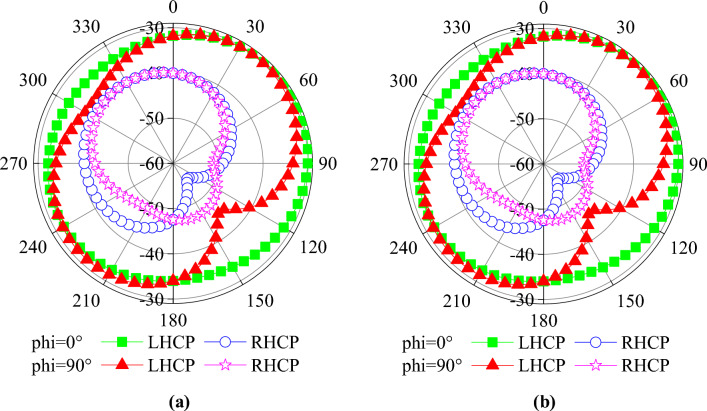
(**a**) Radiation patterns at 0.9 GHz. (**b**) Radiation patterns at 2.45 GHz.

**Figure 10:**
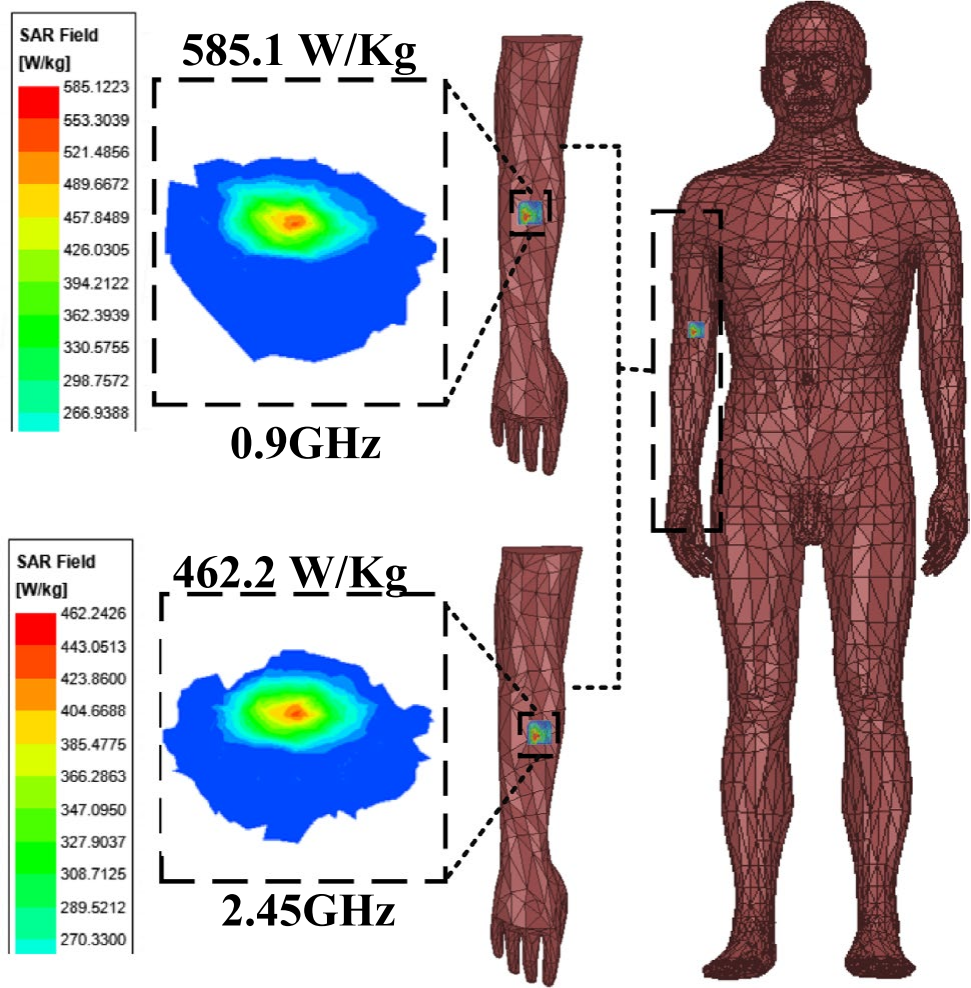
The simulated SAR at 0.9 GHz and 2.45 GHz.

Figure 7a and b shows the simulated current distribution of the antenna at 0.9 and 2.45 GHz. At 0.9 GHz, the currents show a relatively strong concentration on the upper side of the antenna, especially in the region near the circular boundary, and the GND currents are mainly concentrated near the upper side slots. At 2.45 GHz, the cur-rent is more uniformly distributed throughout the circular region and the GND current is uniformly distributed near the cross slot. In addition, for both resonant frequencies, significant current densities can be found near the short-circuit pin locations. The simulated S11 and AR are shown in Fig. 8a and b. The effective 3 dB axial ratio bandwidth covers 0.9 and 2.45 GHz bands in ISM bands very well. The radiation pattern of the antenna is simulated in a single skin model as shown in Fig. 9. The peak gains are -29.5 dBi and -19.2 dBi at 0.9 and 2.45 GHz. As shown in Fig. 9a, the LHCP is more important than the RHCP at 0.9 GHz, so it is LHCP in the lower frequency band. However, at 2.45 GHz, the LHCP and RHCP characteristics are not significant, as shown in Fig. 9b. Specific Absorption Rates (SAR) is a critical performance index of the implantable antenna. The simulated SAR distribution of the implanted antenna designed in this paper in the skin tissue model is shown in Fig. 10. Assuming the transmission power of the antenna is 1W, the maximum 1-g average SAR values at 0.9 GHz and 2.4 GHz are 585.1W/Kg and 462.2W/Kg, respectively. The maximum transmitter power should be less than 2.73mW and 3.46mW to meet the IEEE C95.1–2005 standard^31^.

## Measurement results analysis and communication capability calculation

### Measurement results analysis

**Figure 11:**
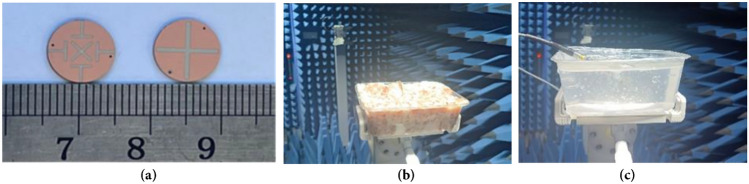
Photograph of the antenna and testing environment. (**a**) The antenna prototype. (**b**) Measured in minced pork. (**c**) Measured in skin gel.

Based on the sizes of the designed antenna in Table 2, a prototype is fabricated and measured, as shown in Fig. 11. And the measurements are carried out by using the Agilent vector network analyzer (PNA-X) and the microwave anechoic chamber developed by the 41st Institute of China Electronics Technology.

**Figure 12:**
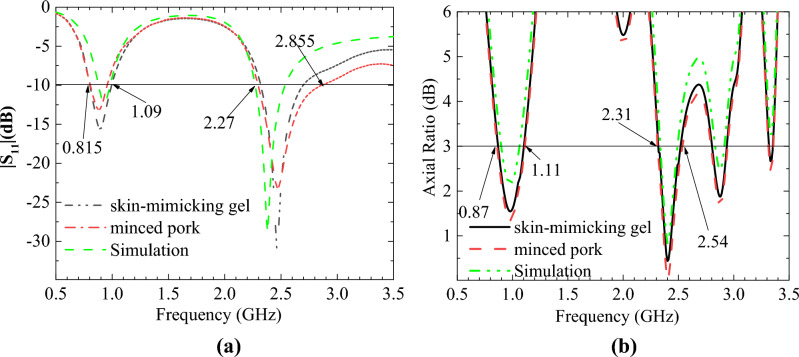
Comparison of the simulated and measured S_11_ and AR in different scenarios. (**a**) S_11_. (**b**) AR.

**Figure 13:**
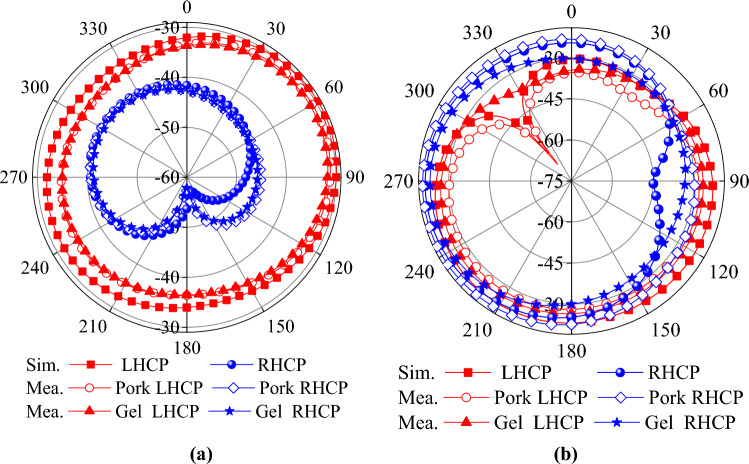
Simulated and measured radiation patterns when phi = 0°. (**a**) 0.9 GHz. (**b**) 2.45 GHz.

The simulated and measured S_11_ and AR are shown in Fig. 12. The simulated and measured S_11_ match each other very well at 0.9 GHz. However, there is a slight deviation between the simulated results and the two measurements at 2.4 GHz. The reason is that the electrical characteristics of the test environment are very close to but not identical to the simulation environment. The radiation patterns are shown in Fig. 13. Good agreement is observed between the antenna’s simulated and measured radiation patterns in the two ISM bands. The slight difference is due to the difference between the simulated and actual test environment and the loss of the RF connecting line.

Table 3 compares the geometric dimensions and important radiation performance indicators of the antenna designed in this article with those in some references. It can be seen that the antenna designed in this article has the smallest physical size and good radiation performance.

**Table 3:** Proposed antenna comparison with references.

Ref	Volume in *λ*_0_ (mm^3^)	Freq. (GHz)	BW (MHz)	Gain (dBi)	CP
^17^	π × 0.024^2^ × 0.0059	1.4	298.2	− 32	Yes
		2.45	521	− 31.6	No
^18^	0.029 × 0.029 × 0.0029	0.86	134	− 31.8	Yes
		1.85	138	− 21.8	
		2.45	458	− 18.5	
^24^	0.081 × 0.084 × 0.0073	2.45	710	− 24.7	Yes
^27^	1.429 × 1.429 × 0.0114	8.6	5630	5.6	Yes
^28^	0.43 × 0.43 × 0.0115	2.17	60	− 23	Yes
		2.38	10		
		3.48	60		
^29^	0.48 × 0.67 × 0.018	6.6	6900	3.2	Yes
Prop	π × 0.014^2^ × 0.0027	0.915	220	− 29.5	Yes
		2.45	230	− 19.2	Yes

### Communication capability calculation

To gain a more comprehensive understanding of the data transmission capability of the antenna, we calculated its link margin (LM) according to formulas (1)–(6) as shown below. Table 4 lists the parameters required for calculation.

1 $$L_{f} = 20\log_{10} \frac{{4\uppi d}}{\lambda }$$

where, d is the distance between Tx and Rx antennas, λ It is the wavelength of the working frequency in free space. LM is used to measure the communication performance of implanted antennas. The calculation formula for LM is

2 $$LM = CNR_{link} - CNR_{required}$$

3 $$CNR_{link} = P_{t} + G_{t} + G_{r} - L_{f} - N_{0}$$

4 $$CNR_{required} = \frac{{E_{b} }}{{N_{0} }} + 10\log_{10} B_{r} - G_{c} + G_{d}$$

5 $$N_{0} = 10\log_{10} k + 10\log_{10} T_{i}$$

6 $$T_{i} = T_{0} (NF - 1)$$

**Table 4:** Link Budget Parameters.

Transmitting Antenna			Receiving antenna		
Frequency(GHz)	0.9	2.45	Frequency(GHz)	0.9	2.45
Power(dBw)	− 46	− 46	Gain(dBi)	2.15	2.15
Gain(dBi)	− 29.5	− 19.2	Polarization	CP	CP
			Noise coefficient(dB)	293	293
			Boltzmann constant k	1.38 × 10^–23^	1.38 × 10^–23^
			N0 (dB/Hz)	− 199.95	− 199.95
Signal Quality					
Frequency(GHz)		0.9		2.45	
Bit rate Br (Mb/s)		1		1	
Error rate		1.0 × 10^–5^		1.0 × 10^–5^	
Eb/N0 (dB)		9.6		9.6	
Encoding gain(dB)		0		0	
Attenuation correction factor Gd (dB)		2.5		2.5	

**Figure 14:**
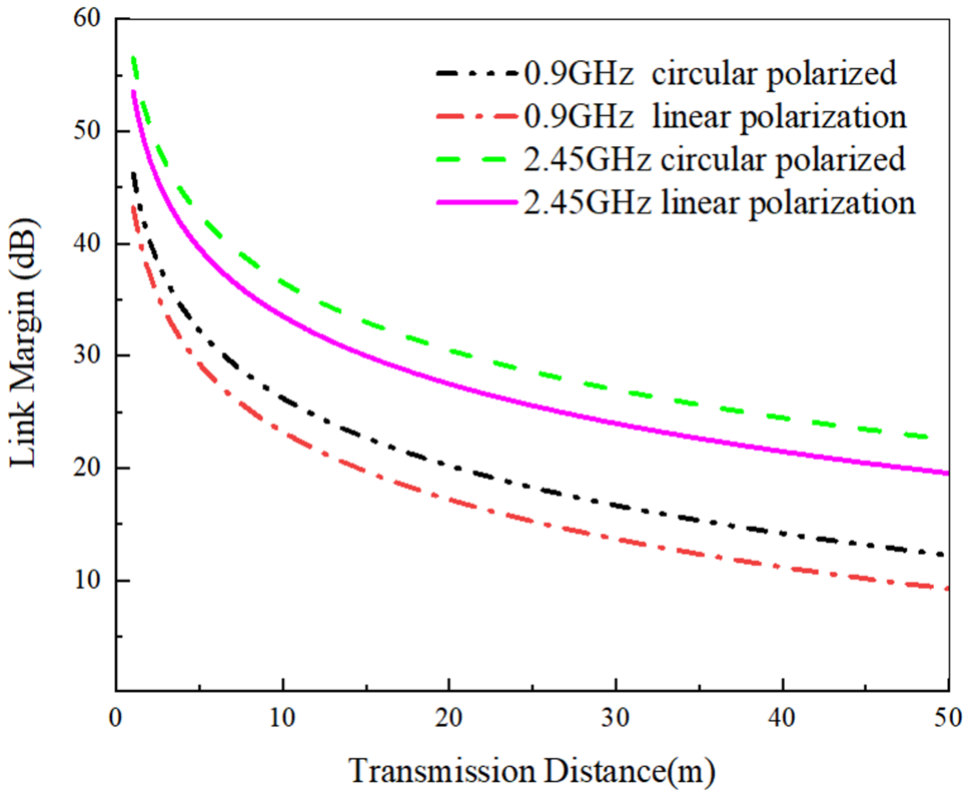
The communication link margin varies with the distance between the transmitting antenna and the receiving antenna.

If the ratio of the signal power received by the external antenna at a specified dis-tance to the noise power density of the implanted antenna transmitted at the specified power is set, then the required CNR is the signal-to-noise ratio required by the carrier receiver to meet a certain communication rate and BER requirements, which is related to the sensitivity of the receiver. Here, we use BPSK modulation, which requires a BER of less than 1 × 10^–5^ and a bit rate of 1 Mb/s Br^32^. The current input power of the antenna operating at 0.9 GHz and 2.4 GHz is − 46 dBm, and the external receiving antenna adopts a circularly polarized antenna with a gain of 2.15 dBi. The calculation result of link margin is shown in Fig. 14. According to the formula, when the receiving antenna is a circularly polarized antenna, the communication distance is approximately 5 m longer than that of a linearly polarized receiving antenna (Table 4).

## Conclusions

A dual-band dual CP implantable antenna is designed by using the eigenmode analysis method in this letter. We have analyzed the working principle of this antenna in detail and obtained a group of better antenna parameters. A prototype is fabricated and measured in skin gel and minced pork. The measured results show that the effective 3 dB AR bandwidth covers the 0.9 GHz and 2.45 GHz bands of ISM very well, and the radiation pattern has good symmetry. Its radius is only 0.0137λ0 (4.7 mm), and λ0 is the wavelength corresponding to the lowest working frequency in free space. It is a good candidate for implantable medical equipment use.

## Data Availability

All data generated or analysed during this study are included in this article.
